# Longitudinal Dysregulation of Adiponectin and Leptin Following Blast-Induced Polytrauma in a Rat Model

**DOI:** 10.3390/ijms26146860

**Published:** 2025-07-17

**Authors:** Rex Jeya Rajkumar Samdavid Thanapaul, Manoj Govindarajulu, Chetan Pundkar, Gaurav Phuyal, Ondine Eken, Joseph B Long, Peethambaran Arun

**Affiliations:** Blast-Induced Neurotrauma Branch, Center for Military Psychiatry and Neuroscience, Walter Reed Army Institute of Research, Silver Spring, MD 20910, USA; rexjeyarajkumar.samdavidthanapaul.ctr@health.mil (R.J.R.S.T.); manoj.y.govindarajulu.ctr@health.mil (M.G.); chetan.y.pundkar.ctr@health.mil (C.P.); gaurav.phuyal.ctr@health.mil (G.P.);

**Keywords:** blast-induced polytrauma, adipokines, leptin, adiponectin, chronic inflammation

## Abstract

Blast-induced polytrauma (BIPT) is a common injury among military personnel exposed to explosive blasts. It is increasingly recognized as a complex, multisystem disorder that extends beyond neurological damage to include systemic metabolic and inflammatory dysfunction. Adipokines, particularly leptin and adiponectin, are hormones secreted by adipose tissue and are emerging as key mediators in the pathophysiology of traumatic brain injuries. Yet, their long-term dynamics following blast exposure remain unclear. This study investigated the temporal profiles of plasma leptin and adiponectin in a longitudinal rat model of BIPT. Adult male Sprague Dawley rats were subjected to either a single (B) or repeated (BB) blast exposure (20 psi) or served as sham controls. Plasma samples were collected at 24 h, 1 month, 6 months, and 12 months post-exposure, and adipokine levels were measured using Enzyme-linked Immunosorbent Assay. Adiponectin levels exhibited a biphasic response: both B and BB groups showed significant early decrease at 24 h and 1 month compared to sham animals, followed by robust elevation at 6 and 12 months, particularly in the repeated blast group. In contrast, leptin levels remained unchanged acutely but rose significantly at 6 and 12 months post-blast, with the BB group again showing the highest levels. These patterns indicate sustained, exposure-dependent dysregulation of adipokine signaling after blast trauma. The study provides the first longitudinal profile of systemic adipokine responses to BIPT, revealing their potential as accessible biomarkers and therapeutic targets. These findings support a model of chronic metabolic and inflammatory imbalance in BIPT and warrant further investigation in human cohorts and mechanistic studies.

## 1. Introduction

Blast-induced polytrauma (BIPT), a hallmark injury of modern warfare, arises from exposure to explosive blasts and poses complex long-term health challenges for military personnel and veterans [1]. Although BIPT may initially present with acute symptoms such as headaches, dizziness, and disorientation, it often advances to chronic conditions including cognitive impairment, post-traumatic stress disorder (PTSD), and neurodegenerative diseases [2]. The pathophysiology of BIPT is multifactorial, encompassing primary blast waves that directly impact various tissues, along with secondary and tertiary injuries resulting from projectiles and bodily displacement, respectively [3]. These trauma mechanisms lead to widespread neurological and systemic consequences such as traumatic brain injury (TBI), chronic inflammation, and metabolic dysregulation [4].

Emerging evidence suggests a central role for adipokines—hormones predominantly secreted by adipose tissue such as leptin and adiponectin—in modulating the metabolic and inflammatory cascades associated with BIPT [5,6]. Leptin, known for its roles in energy homeostasis, appetite regulation, and neuroendocrine signaling, has also been implicated in pro-inflammatory pathways [7]. In contrast, adiponectin is recognized for its anti-inflammatory, anti-apoptotic, and neuroprotective effects [8]. Dysregulation of these adipokines has been associated with TBI and various neurodegenerative conditions [6]; however, their specific roles in blast-induced injury remain insufficiently explored.

Military personnel exposed to blast events often experience persistent systemic inflammation and metabolic dysfunction [9], conditions that are frequently worsened by imbalanced adipokine levels. These disruptions are linked to secondary comorbidities such as obesity, diabetes, cardiovascular disease, and accelerated cognitive decline [10]. Organs sensitive to adipokine signaling, including the brain, liver, and adipose tissue, are particularly susceptible to trauma-induced dysfunction [11,12,13,14,15]. Furthermore, factors such as elevated body mass index (BMI), poor nutrition, chronic stress, and physical inactivity may further influence adipokine levels, exacerbating post-traumatic complications [11].

Blast exposure is increasingly recognized not only for its acute neurological impact but also for its contribution to long-term systemic metabolic and inflammatory disturbances. While the blast itself triggers an immediate neuroimmune response and initiates adipokine dysregulation, secondary lifestyle changes following injury, such as reduced physical activity, chronic stress, disrupted sleep, and poor diet, may further exacerbate these effects during the chronic phase of recovery. These indirect factors are highly relevant in military populations, where veterans with blast exposure often exhibit metabolic abnormalities, obesity, and hormonal imbalance over time. For instance, longitudinal studies have shown that post-9/11 veterans with blast exposure (with or without TBI) are more likely to develop metabolic syndrome and adipokine-related disturbances, including altered BMI trajectories and systemic inflammation [16,17]. Additionally, chronic adipose redistribution and hypothalamic pathology have been reported in animal models of blast trauma, supporting mechanistic links between central injury and peripheral metabolic changes [18]. These findings suggest that lifestyle and neuroendocrine factors may interact with blast-induced pathophysiology to perpetuate a long-term imbalance in adipokines. Thus, while the current study focuses on primary blast effects, the chronic dysregulation observed may be amplified by secondary psychosocial and behavioral changes commonly encountered in affected individuals [19].

Blast exposure may disrupt the physiological balance of leptin and adiponectin, which can worsen neuroinflammation and lead to systemic metabolic dysregulation [18]. Despite the biological significance of these adipokines, there is a shortage of longitudinal studies that characterize their dynamic changes after blast trauma, particularly under military-relevant conditions. A longitudinal study carried out in rats revealed that blast exposure induces severe neurobehavioral changes at 6 and 12 months post-blast, indicating that chronic problems after blast exposure are more severe than the acute changes [20]. Thus, studies focusing on the changes in adipokines at chronic time points post-blast are warranted.

The increasing prevalence of explosive weaponry in modern conflicts highlights the urgent need to understand the systemic effects of blast exposure, particularly the long-term metabolic and inflammatory consequences that may not be immediately apparent. Adipokines such as leptin and adiponectin, known for their roles in energy homeostasis and inflammation, have been implicated in the body’s response to traumatic injuries. Investigating the dysregulation of these adipokines following blast exposure can provide insights into the chronic health issues faced by affected populations and inform potential therapeutic strategies. Considering this gap in the research, this study aimed to investigate the relationship between blast exposure and the time-dependent changes in leptin and adiponectin levels, which impact both systemic and neurological outcomes in BIPT. Using a rat model of BIPT, we aim to investigate the temporal profile of plasma adipokine levels to connect their potential role in post-blast metabolic and inflammatory disruptions. The insights gained from this study may help identify new biomarkers or therapeutic targets, ultimately guiding the development of strategies to reduce the long-term consequences of blast injuries and enhance health outcomes for affected military personnel and veterans.

## 2. Results

To investigate the systemic metabolic and inflammatory consequences of BIPT, plasma levels of two key adipokines, adiponectin and leptin, were measured at 24 h, 1 month, 6 months, and 12 months post-exposure in rats assigned to sham (control), single blast (B), or repeated blast (BB) groups. Two-way ANOVA revealed significant main effects of group, time, and their interaction for both adipokines (*p* < 0.001), indicating dynamic and exposure-dependent changes.

**Figure 1:**
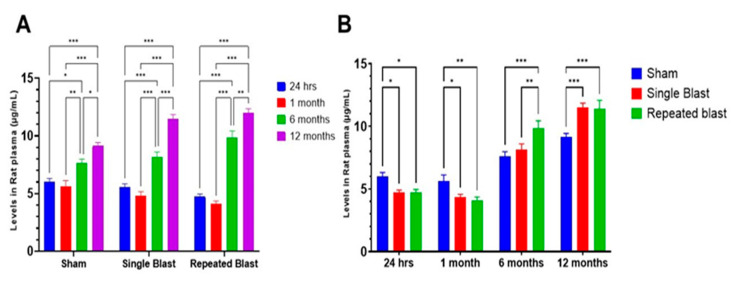
**Plasma adiponectin levels with aging and following blast exposure.** (**A**) Changes in plasma adiponectin concentrations with age in sham, single blast (**B**), and repeated blast (BB) groups. Adiponectin values for each time point were compared to the other time points evaluated. Data are presented as mean ± SEM. * *p* < 0.05, ** *p* < 0.01, *** *p* < 0.001. (**B**) Temporal analysis of plasma adiponectin levels across all groups at 24 h, 1 month, 6 months, and 12 months post-blast. Data are presented as mean ± SEM. * *p* < 0.05, ** *p* < 0.01, *** *p* < 0.001; *n* = 10–12 per group.

Adiponectin levels displayed a biphasic response to blast exposure (Figure 1A,B). At 24 h, both the single blast (4.71 µg/mL) and repeated blast (4.74 µg/mL) groups exhibited significantly reduced adiponectin levels compared to the sham group (6.0 µg/mL) (*p* < 0.05), indicating an early-phase suppression. This suppression persisted at 1 month, where levels in the single (4.36 µg/mL) and repeated (4.09 µg/mL) blast groups remained significantly lower than in sham animals (5.61 µg/mL) (*p* < 0.05 and *p* < 0.01, respectively). By 6 months, adiponectin levels reversed course and increased significantly in both blast groups: 8.16 µg/mL in the single blast and 9.86 µg/mL in the repeated blast group, compared to 7.63 µg/mL in the sham (*p* < 0.001 and *p* < 0.01, respectively). At 12 months, this trend intensified, with adiponectin levels reaching 11.49 µg/mL in the single and 11.40 µg/mL in the repeated blast groups, respectively, both significantly higher than in sham controls (9.31 µg/mL) (*p* < 0.001). Repeated blast exposure consistently resulted in the most pronounced changes, especially during the chronic phase, with values exceeding those in both the sham and single blast groups (*p* < 0.05 to *p* < 0.001). The sham group also exhibited a modest, age-related increase in adiponectin from 5.99 µg/mL at 24 h to 7.63 µg/mL at 6 months and 9.31 µg/mL at 12 months (*p* < 0.05 to *p* < 0.001). This trend is consistent with previously reported age-related physiological changes in adiponectin, particularly under conditions of controlled dietary intake and stable body weight. Similar findings were reported [21], where age-related increases in plasma adiponectin were observed in rats undergoing long-term caloric restriction, providing a physiological baseline that reinforces the validity of age-matched comparisons with blast-exposed groups. In contrast, the blast-exposed animals displayed a biphasic adiponectin response, initially decreasing during the acute phase and then increasing significantly later, especially after repeated blast exposure. This pattern suggests ongoing dysregulation of adiponectin signaling, which could reflect a delayed compensatory response or a chronic pathological adaptation to blast-induced systemic stress.

**Figure 2:**
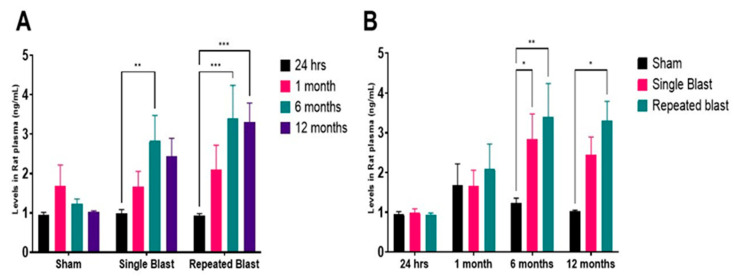
**Plasma leptin levels with aging and following blast exposure.** (**A**) Changes in plasma leptin concentrations with age in sham, single blast (**B**), and repeated blast (BB) groups. Leptin values for each time point were compared to the other time points evaluated. Data are expressed as mean ± SEM. ** *p* < 0.01, *** *p* < 0.001. (**B**) Temporal analysis of plasma leptin levels across all groups at 24 h, 1 month, 6 months, and 12 months post-blast. Data are expressed as mean ± SEM. * *p* < 0.05, ** *p* < 0.01; *n* = 10–12 per group.

Leptin levels remained stable and low during the acute phase after blast exposure (Figure 2A,B). At 24 h, there were no significant differences between groups (sham: 0.94 ng/mL; single blast: 0.98 ng/mL; repeated blast: 0.92 ng/mL). By 1 month, a modest increase was noted in the BB group (sham: 1.68 ng/mL; single blast: 1.66 ng/mL; repeated blast: 2.1 ng/mL), though not statistically significant. A substantial rise in leptin levels occurred at 6 months, with the single blast group reaching 2.83 ng/mL (*p* < 0.01 vs. sham) and the repeated blast group reaching 3.40 ng/mL (*p* < 0.001 vs. both sham and single blast groups). At 12 months, leptin remained elevated in both blast groups (single blast: 2.44 ng/mL; repeated blast: 3.3 ng/mL), significantly exceeding sham (1.0 ng/mL) (*p* < 0.001). Although a plateau relative to 6 months values was observed, levels remained chronically elevated at 12 months post-blast. These findings reveal a delayed but sustained leptin upregulation following blast exposure, with repeated blasts yielding the most pronounced elevation. This time- and number of blast exposure-dependent pattern suggests that leptin may serve as a biomarker of chronic inflammation or metabolic disturbance linked to BIPT.

The data demonstrate that adiponectin and leptin undergo distinct yet complementary changes following blast exposure at chronic time points. Adiponectin exhibits a biphasic pattern, characterized by early suppression and chronic elevation, while leptin displays a delayed and sustained increase, particularly in the repeated blast group. These results underscore the long-term dysregulation of adipokine signaling after blast injury, highlighting their potential as biomarkers or therapeutic targets in the context of chronic inflammation and metabolic dysfunction associated with BIPT.

## 3. Discussion

This study presents the first comprehensive longitudinal investigation of adipokine dysregulation—specifically, adiponectin and leptin—in a rat model of BIPT. Our findings reveal sustained number of exposure-dependent alterations in these key metabolic and inflammatory regulators following both single and repeated blast exposures. These data build upon prior observations and provide mechanistic insight into the systemic metabolic and neuroimmune responses triggered by blast trauma, with direct translational relevance for military personnel and veterans.

These findings highlight distinct and time-dependent patterns of adipokine dysregulation, suggesting that blast exposure initiates a prolonged state of metabolic and inflammatory imbalance. The biphasic suppression and rebound of adiponectin, coupled with the delayed yet sustained elevation in leptin, reflect complex homeostatic adaptations likely driven by neuroimmune and neuroendocrine disturbances. In particular, alterations in hypothalamic–pituitary–adrenal (HPA) axis activity, sympathetic tone, and peripheral cytokine signaling may contribute to the observed temporal dynamics. These systemic changes echo clinical findings in blast-exposed veterans, who often present with long-term endocrine dysfunction, chronic inflammation, and metabolic syndrome.

Research on the specific connection between blast exposure and adiponectin levels is limited. In our study, plasma adiponectin levels exhibited a biphasic pattern, characterized by early suppression (24 h–1 month) followed by sustained elevation at 6 and 12 months, particularly in the BB group. A previous study carried out in rats revealed no significant changes in plasma levels of adiponectin at 1 or 3 months after a single blast at 17 psi, but that study did not explore the changes occurring at chronic time points like 6 or 12 months post-blast [18]. But mRNA analysis conducted in those rats indicated that *adiponectin* expression was significantly higher in the abdomen and lower in the lower jaw at 3 months post-blast [18]. Rats subjected to lateral fluid percussion injury showed decreased plasma adiponectin levels at 48 h and 72 h post-injury [22]. Similarly, in a mouse model of transient focal cerebral ischemia, plasma adiponectin levels increased transiently up to 3 h after reperfusion, and decreased (by 60%) thereafter in a time-dependent manner up to 48 h after reperfusion [23]. In patients with acute intracerebral hemorrhage and acute ischemic stroke, serum adiponectin levels were lower in patients at admission compared with healthy volunteers [24]. Furthermore, lower adiponectin values were observed in stroke patients before neurorehabilitation in comparison to controls [25]. Interestingly, in TBI patients, higher serum adiponectin levels during the acute phase of TBI has been shown to be associated with and independently predictive of favorable outcomes [26]. Thus, it is hypothesized that accumulation (recruitment) of adiponectin in the brain might contribute to the decrease in the adiponectin levels in the peripheral blood and this process might also be associated with cerebroprotective effect of adiponectin [22]. The increase in plasma adiponectin levels at 6–12 months likely reflects an endogenous attempt to counteract chronic inflammation and metabolic stress, as observed in TBI and neurodegenerative disorders [27]. However, this compensatory response may be inadequate or dysfunctional as chronic stress and oxidative damage may impair adiponectin receptor signaling, reducing its protective effects [28].

Leptin has been suggested as a new biomarker predictive of the severity or outcome of brain injury. For instance, plasma leptin levels of patients with cerebral hemorrhage were found to be substantially higher than in healthy controls [29]. Furthermore, a significant association between leptin levels and clinical outcomes at 6 months was detected [30]. In our study, leptin levels were significantly elevated chronically following blast exposure, with the most pronounced increase observed in the repeated blast group at 6 and 12 months, suggesting a delayed inflammatory response. Interestingly, we did not see any changes in the levels of leptin at 24 h or 1 month post-blast exposure. This delay in leptin increase could be attributed to the acute post-blast stress response and, specifically, acute increases in the sympathetic outflow. Studies indicate that dysautonomia and sympathetic storms are well known to occur after TBI and adrenergic stimulation downregulate leptin expression and secretion by adipocytes [31,32]. Furthermore, normal-to-reduced leptin levels at acute stages following injury may be due to the fasting conditions in the injured patient or animal [33]. After a few months following injury, features of dysautonomia begin to resolve and at this point, it is possible that sympathetic inhibition of leptin signaling gives way to increase in leptin synthesis as observed after TBI unrelated to blast exposure [34,35]. Hence, it is possible that the abatement of sympathetic control on leptin occurred several months following injury considering the complex injury mechanisms related to blast exposure. In a previous study conducted on rats, serum leptin levels were significantly elevated at 24 h following a single blast overpressure (30 psi) exposure but showed reduced serum leptin levels when the rats were exposed to blast three times (days 1, 4, and 7) [36]. Rats subjected to experimental controlled cortical impact injury along with femoral fracture had elevated serum leptin levels at 2 weeks and persisted up to 8 weeks post-insults in comparison to sham controls [37]. In contrary, rats exposed to single overpressure wave (17 psi) showed increased serum leptin hormone levels at 1 month and then reduced levels at 3 months following blast exposure [18]. In those rats, mRNA levels of *leptin* in the abdomen showed significant increase at 3 months post-blast, with the time point showing decreased plasma leptin levels [18].

Mechanistically, blast trauma may trigger leptin production via interleukin-6 (IL-6) and tumor necrosis factor-α (TNF-α) signaling as well as dysregulation of the hypothalamic–pituitary–adrenal (HPA) axis, a known modulator of metabolic and neuroimmune balance [38]. The sustained elevation of leptin at 6 and 12 months indicates persistent low-grade inflammation, a hallmark of chronic BIPT sequelae. The neurobehavioral functional studies carried out in the rats used for the current study showed significant functional deficits at 6 and 12 months post-blast [20]. Leptin’s dual function—as both a neuroprotective hormone and a pro-inflammatory cytokine-like factor—may help explain its complex role. While transient increases may be adaptive, chronic overproduction can be linked to neuroinflammation, insulin resistance, and cognitive decline. Our findings echo previous reports in TBI patients and blast-exposed veterans, where chronic leptin elevation was associated with PTSD, obesity, and metabolic syndrome [39]. Our data support a trajectory from acute response to chronic dysfunction, revealing that metabolic disruption persists long after the initial blast event. These findings also suggest a possible link to accelerated aging phenotypes, consistent with emerging models of trauma-induced premature senescence.

The observed co-elevation of leptin and adiponectin is unusual, as these hormones are generally inversely correlated under normal physiological conditions. This indicates independent dysregulation of metabolic-inflammatory axes and disruption of homeostatic feedback mechanisms. Repeated blast exposure consistently led to greater dysregulation of both adipokines than a single exposure, supporting the hypothesis of cumulative injury. This aligns with clinical observations where military personnel with multiple blast exposures show more severe metabolic, cognitive, and psychological impairments [40,41]. The additive nature of repeated trauma highlights the importance of monitoring and intervention in high-risk individuals.

Additional parallels can be drawn between studies of PTSD and Alzheimer’s disease, where an imbalance of adipokines contributes to neurodegeneration, cognitive impairment, and systemic inflammation [42]. These similarities further support the translational relevance of our findings and their implications for long-term care in trauma-exposed populations. The distinct and sustained patterns of leptin and adiponectin dysregulation highlight their potential as biomarkers for BIPT severity and progression. Longitudinal monitoring of these adipokines could enable early diagnosis and risk stratification. Furthermore, targeted modulation of adipokine signaling, such as leptin and/or adiponectin receptor agonists, may provide novel therapeutic strategies [43,44]. Lifestyle interventions—such as diet, exercise, and stress reduction—that influence adipokine profiles [45] should also be examined in human trials. Pharmacological strategies designed to restore metabolic balance could be used to reduce long-term complications, particularly in individuals with repeated blast exposures.

While this study specifically focused on adiponectin and leptin due to their well-understood and opposing roles in metabolic and inflammatory regulation, we recognize that the adipokine family includes a broader range of mediators relevant to trauma-induced systemic dysfunction. Emerging adipokines such as resistin, chemerin, visfatin, omentin, and lipocalin-2 have been linked to regulating inflammation, insulin resistance, and neuroimmune interactions. Therefore, our ongoing and future studies will use multiplex profiling to achieve a more comprehensive understanding of adipokine dysregulation in the context of BIPT. This broader approach will offer greater insight into adipokine crosstalk and its role in chronic inflammation, metabolic disruption, and behavioral outcomes following blast exposure.

Despite the strengths of this study, including its longitudinal design, controlled blast exposures, and analysis of systemic adipokine dynamics, several limitations should be acknowledged. First, the study focused solely on plasma adipokine levels and did not evaluate tissue-specific expression in key organs, such as the brain, liver, or adipose tissue. While plasma-based ELISA measurements offer translational value as minimally invasive biomarkers, they do not capture local regulatory activity. To address this, we plan to include a follow-up study to investigate spatial regulation and downstream signaling pathways. These tissue-level investigations will help differentiate peripheral from central contributions to adipokine dysregulation. Second, this study used only male rats to reduce variability related to the estrous cycle and hormonal fluctuations. While this helped establish baseline trajectories of adipokine changes after blast exposure, it limits the broad applicability of the findings. Because sex hormones are known to influence adipokine signaling and neuroinflammatory responses, including those in female rats, it is crucial to assess sex-specific vulnerabilities and treatment responses to BIPT in future studies. Third, several physiological and biochemical variables that influence adipokine dynamics, such as food intake, circulating cytokines (IL-6, TNF-α), and oxidative stress markers, were not measured in the present study. Although all animals were maintained on identical diets and feeding schedules to control for metabolic confounders, additional profiling of inflammatory cytokines and metabolic regulators is planned. We will apply multiplex cytokine analysis in future works to better understand the mechanistic links between systemic inflammation and alterations in adipokines. Finally, although efforts were made to control for age-related variability through an age-matched design, standardized housing, and post-blast weight regulation, some biological aging effects over the 12 months are unavoidable. The consistent, physiologically expected increase in adiponectin levels observed in sham animals supports the internal consistency of the model. Nevertheless, we acknowledge that natural aging may have contributed to some of the hormone fluctuations observed and consider this as a limitation of the study.

## 4. Conclusions

This study provides compelling evidence that blast exposure induces chronic, systemic dysregulation of the adipokines leptin and adiponectin, which are key regulators of metabolic and inflammatory homeostasis. Utilizing a longitudinal rat model of BIPT, we demonstrate that both single and repeated blast exposures disrupt normal adipokine dynamics, with repeated exposure causing more pronounced and persistent alterations. Adiponectin showed initial suppression followed by sustained elevation at chronic time points. Meanwhile, leptin exhibited a delayed but prolonged increase, consistent with the development of a chronic low-grade inflammatory state. These findings support the idea of BIPT as a multisystem disorder that extends beyond central nervous system injury to include systemic metabolic and immune dysfunction. The sustained, dose-dependent dysregulation of adipokines observed here may contribute to the long-term consequences commonly reported in blast-exposed individuals, such as neurodegenerative disease, metabolic syndrome, and PTSD. The translational implications of these results are significant. Leptin and adiponectin are accessible, blood-based biomarkers with established roles in both central and peripheral inflammation. Their sustained dysregulation in this model of blast-induced polytrauma suggests potential utility in monitoring disease progression, predicting chronic outcomes, or guiding therapeutic strategies. Future work will include multi-omics and tissue-level analyses to further elucidate underlying mechanisms and validate these markers in clinical settings. Importantly, leptin and adiponectin could serve as accessible, non-invasive biomarkers for BIPT progression and represent novel targets for therapeutic intervention. This study establishes a foundation for a system-level understanding of blast trauma, linking neuroinflammation with peripheral metabolic disruption. Future research should concentrate on mechanistic studies to clarify the pathways underlying adipokine dysregulation, validate these biomarkers in human cohorts, and assess pharmacological or lifestyle interventions aimed at restoring adipokine balance. Ultimately, these efforts will be essential for alleviating the long-term health burden of blast trauma in both military and civilian populations.

## 5. Methods

### 5.1. Animal Use and Ethical Approval

All animal procedures were conducted in compliance with the Animal Welfare Act and the Guide for the Care and Use of Laboratory Animals [46], under a protocol approved by the Institutional Animal Care and Use Committee (IACUC) at the Walter Reed Army Institute of Research (WRAIR). All research took place in an AAALAC International-accredited facility and adhered to applicable federal statutes and regulations governing the ethical treatment of animals in research.

### 5.2. Animal Cohorts, Housing, and Diet Regulation

A total of 125 adult male Sprague Dawley rats (7–8 weeks old, 250–275 g) were obtained from Charles River Laboratories (MA, USA). Animals were housed in individually ventilated cages under standardized environmental conditions (temperature: 20–22 °C; 12 h light/dark cycle) with uniform bedding. All rats had ad libitum access to food and water until one month after blast exposure. To reduce variability due to aging and housing throughout the 12-month study, sham animals were maintained in parallel with blast-exposed groups at each time point (24 h, 1 month, 6 months, 12 months), allowing for direct age-matched comparisons. From one month post-exposure onward, all animals were placed on a restricted feeding regimen to maintain body weights between 450 and 500 g, thereby minimizing obesity-related metabolic variation. Only male Sprague Dawley rats were used in this study to reduce variability associated with estrous cycle fluctuations, which are known to influence adipokine levels and inflammatory responses. This approach is consistent with previous preclinical studies of blast-induced traumatic brain injury (bTBI) and was selected to establish a clear baseline for longitudinal adipokine profiling following blast-induced polytrauma (BIPT). Animals were fed a standard rodent chow (Prolab IsoPro RMH 3000; LabDiet, St. Louis, MO, USA), which contained 22% protein, 5% fat, and 4.4% fiber, with a metabolizable energy content of approximately 3.4 kcal/g. No high-fat or specialized diets were used at any point during the study. Identical dietary composition and feeding schedules were applied across all groups to ensure that any observed differences in adipokine levels were attributable solely to blast exposure rather than nutritional factors.

### 5.3. Experimental Grouping and Randomization

Rats were randomly assigned to one of three groups: sham (control), single blast (B), or repeated blast (BB). Stratified randomization was used to ensure balanced distribution of body weight and health status across groups. Each group included 10 to 12 independent biological replicates per time point (24 h, 1 month, 6 months, and 12 months), yielding a total of 125 animals. Separate cohorts were used for each time point to maintain statistical independence and minimize biological variability. The selected group sizes were based on power calculations from previous blast injury studies and were sufficient to detect significant differences in plasma adipokine levels.

### 5.4. Blast Exposure

Blast exposures were conducted using a calibrated Advanced Blast Simulator (ABS), as previously described [20]. Rats were anesthetized with 5% isoflurane in air for less than 8 min to minimize physiological variability. Animals were placed in the prone position and aligned longitudinally within the ABS. A blast overpressure of approximately 20 psi was selected based on prior validated studies at WRAIR, where this intensity consistently produced mild-to-moderate bTBI in rodents, accompanied by reproducible behavioral and neuropathological changes relevant to military-relevant trauma. The single blast group (B) received one ~20 psi overpressure wave, while the repeated blast group (BB) received two tightly coupled ~20 psi waves separated by a 2 min interval to simulate repeated exposure scenarios. Sham animals underwent the same anesthesia and handling without blast exposure. The ABS was recalibrated prior to each session to ensure consistent delivery of overpressure waveforms. All downstream analyses were performed by researchers blinded to group assignments.

### 5.5. Blood Collection and Plasma Preparation

At designated time points (24 h, 1 month, 6 months, 12 months), rats were euthanized by isoflurane overdose (5%) followed by cardiac puncture. Whole blood was collected into EDTA-coated BD Vacutainer tubes (Becton, Dickinson and Company, Franklin Lakes, NJ, USA), centrifuged at 3000 rpm for 15 min at 4 °C. Plasma was aliquoted and stored at −80 °C until analysis. Baseline (pre-blast) plasma was not collected due to logistical constraints and blood volume limitations; however, randomization was used to minimize inter-group variability.

### 5.6. ELISA for Adipokine Quantification

Plasma levels of leptin and adiponectin were measured using rat-specific, enzyme-linked immunosorbent assay (ELISA) kits from Crystal Chem (Elk Grove Village, IL, USA). The Rat Leptin ELISA Kit (Catalog #90040) had a standard curve range of 0.2–20 ng/mL, sensitivity of 0.04 ng/mL, and intra-assay coefficient of variation (CV) <5%. The Rat Total Adiponectin ELISA Kit (Catalog #80570) had a standard curve range of 0.5–30 µg/mL, sensitivity of 0.15 µg/mL, and intra-assay CV <6%. All samples were analyzed in duplicate, and values were averaged to reduce technical variability. Assay specificity and reproducibility were validated according to the manufacturer’s instructions.

### 5.7. Statistical Analysis

Data are presented as mean ± standard error of the mean (SEM). Statistical comparisons were performed using two-way analysis of variance (ANOVA), followed by Tukey’s post hoc test for multiple comparisons. A *p*-value of <0.05 was considered statistically significant. Analyses were performed using GraphPad Prism version 9.5.1.733 (GraphPad Software, Solana Beach, CA, USA).

## Data Availability

Data are within the communication.

## Disclaimer

Material has been reviewed by the Walter Reed Army Institute of Research. There is no objection to its presentation and/or publication. The opinions or assertions contained herein are the private views of the author, and are not to be construed as official, or as reflecting the true views of the Department of the Army or the Department of Defense. Research was conducted under an IACUC-approved animal use protocol in an AAALAC International-accredited facility with a Public Health Services Animal Welfare Assurance and in compliance with the Animal Welfare Act and other federal statutes and regulations relating to laboratory animals.
